# Supplementary material for: Empirical methods for controlling false positives and estimating confidence in ChIP-Seq peaks
Source: BMC Bioinformatics. 2008 Dec 5;9:523. doi: 10.1186/1471-2105-9-523 (PMC2628906; doi:10.1186/1471-2105-9-523)
Supplement: Additional File 1 — A variety of html documents from the USeq web site detailing the available applications, their best usage, output file type descriptions, command line menus, etc. [file 1471-2105-9-523-S1.zip › USeqUserGuides/JavaDoc.zip!/JavaDoc/trans/tpmap/package-use.html]

Uses of Package trans.tpmap


|  |  |  |
| --- | --- | --- |
| **Overview** \| **Package** \| Class \| **Use** \| **Tree** \| **Deprecated** \| **Index** \| **Help** | |  |
| PREV   NEXT | **FRAMES**    **NO FRAMES**     **All Classes** |  |


---


## **Uses of Package trans.tpmap**

| Packages that use trans.tpmap | |
| --- | --- |
| **trans.anno** | For associating intervals, binding regions, and binding peaks with GFF annotation. |
| **trans.cel** | Apps and methods for manipulating cel files. |
| **trans.tpmap** | For maping, filtering, and calculating statistics about text version bpmap files. |

Classes in trans.tpmap used by trans.anno

****TPMapLine****
  
          Minimal tpmap line, chrom, start, nothing else.

Classes in trans.tpmap used by trans.cel

****IntensityFeature****
  
          Minimal info about a feature for combining multiple chips together.

****MapFeature****
  
          Tpmap line broken into some components, chromosome, start, pmX, pmY, and optionally, mmX, mmY.

Classes in trans.tpmap used by trans.tpmap

****BlastFilterResult****
  
          Container for a blast result: chromosome, numMatches, exactMatches, oneBPMismatches, startExactMatch.

****MapFeature****
  
          Tpmap line broken into some components, chromosome, start, pmX, pmY, and optionally, mmX, mmY.

****TPMapFeature****
  
          Container for info about a particular oligo feature from a tpmap file.

****TPMapLine****
  
          Minimal tpmap line, chrom, start, nothing else.

---


|  |  |  |
| --- | --- | --- |
| **Overview** \| **Package** \| Class \| **Use** \| **Tree** \| **Deprecated** \| **Index** \| **Help** | |  |
| PREV   NEXT | **FRAMES**    **NO FRAMES**     **All Classes** |  |


---
